# Malaria and Typhoid Fever Coinfection Among Febrile Patients Attending a Health Facility in Yaoundé, Cameroon: A Cross-Sectional Study

**DOI:** 10.1155/japr/9977704

**Published:** 2025-11-12

**Authors:** Palmer Masumbe Netongo, Ange Maxime Tchoutang, MacDonald Bin Eric, Arnaud Tepa, Marie Christine Nzuno, Severin Donald Kamdem

**Affiliations:** ^1^Molecular Diagnostics Research Group, Biotechnology Centre, University of Yaoundé 1, Yaoundé, Cameroon; ^2^Department of Biochemistry, University of Yaoundé 1, Yaoundé, Cameroon; ^3^School of Science, Navajo Technical University, Crownpoint, New Mexico, USA; ^4^Laboratory for Public Health Research Biotechnologies, University of Yaoundé 1, Yaoundé, Cameroon; ^5^Faculty of Medicine and Biomedical Sciences, University of Yaoundé 1, Yaoundé, Cameroon; ^6^Center for Research on Infectious Diseases (CRID), Yaoundé, Cameroon; ^7^Department of Biochemistry, Faculty of Science, University of Buea, Buea, Cameroon; ^8^School of Health Sciences, Catholic University of Central Africa, Yaoundé, Cameroon

**Keywords:** Cameroon, malaria, prevalence, risk factors, typhoid fever

## Abstract

**Background:**

Malaria and typhoid fever are significant public health challenges in tropical regions, with Cameroon serving as a notable example of their endemicity. This study is aimed at determining the prevalence of malaria, typhoid fever, and their coinfection and assessing associated risk factors among febrile patients attending a tertiary hospital.

**Methodology:**

A cross-sectional study was conducted between June and August 2023 at the Etoug-Ebe Baptist Hospital in Yaoundé, Cameroon. Two hundred and eighty-eight febrile patients aged over 2 years, suspected of having malaria and/or typhoid fever, were enrolled after obtaining informed consent from adults or a legal representative for minors. Data were collected using a structured case report form (CRF). Malaria diagnosis involved a rapid diagnostic test (RDT) for screening, followed by confirmation with thick blood smear microscopy. For typhoid fever diagnosis, a combination of serological testing (Widal and IgM/IgG rapid tests) and rapid stool antigen detection (for *Salmonella* O and H antigens) was used. Data analysis was conducted using SPSS Version 26 and GraphPad Prism Version 9 software.

**Results:**

Out of the 288 participants, 179 (62.5%) tested positive for malaria and/or typhoid fever. According to our diagnostic algorithm, the prevalence rates for malaria, typhoid fever, and coinfection were 44.79% (*n* = 129), 37.15% (*n* = 107), and 19.79% (*n* = 57), respectively. Coinfection was significantly associated with fever (*χ*^2^ = 7.092, *p* = 0.008) and profuse sweating (*χ*^2^ = 3.774, *p* = 0.05), while jaundice was linked to typhoid fever (*χ*^2^ = 4.777, *p* = 0.029). Notable risk factors for malaria and typhoid fever included the presence of ponds of water and/or clumps of grass around the house and the main source of drinking water.

**Conclusion:**

Malaria and typhoid fever were prevalent among febrile patients in Yaoundé, with malaria emerging as the leading cause and notable levels of coinfection. Clinical symptoms and environmental risk factors were associated with disease occurrence, underscoring the need for strengthened control strategies.

## 1. Introduction

Fever is one characteristic of the host's response to a variety of infections [1]. In the tropics and subtropics, it remains the main source of consultation, particularly for two diseases: malaria and typhoid fever [2], and both diseases are associated with poverty and underdevelopment [3]. Malaria is one of the prominent causes of death in Africa, especially in children under the age of 5 years and pregnant women [4]. It is caused by a protozoan parasite of the Apicomplexa phylum called *Plasmodium*, transmitted by the bite of an anopheles mosquito during a blood meal. Five (5) species of *Plasmodium* are responsible for malaria: *Plasmodium falciparum* (Pf) (accounting for most deaths), *Plasmodium vivax*, *Plasmodium ovale*, *Plasmodium malariae*, and *Plasmodium knowlesi* [5]. According to the World Health Organization, there were an estimated 249 million cases in the world in 2022, and the majority of cases are from Africa [6]. In Cameroon, malaria is the most widespread endemic disease and is responsible for the majority of absenteeism from school. There were over 2.7 million cases and 12,600 reported deaths in 2022, with Cameroon counted among the 15 highest burden malaria countries [6].

Typhoid fever, on the other hand, remains the predominant enteric fever in the world [7]. Four (4) species play a key role in the development of the disease, and they are *Salmonella typhi*, along with three related strains *Salmonella paratyphi A*, *B*, and *C* [8]. Typhoid fever is transmitted by ingestion of contaminated food and water via the fecal–oral route [9]. It is estimated that between 11 and 21 million cases and approximately 128,000 to 161,000 typhoid-related deaths occur annually [10]. The majority of cases occur in South/Southeast Asia and sub-Saharan Africa [11]. Higher fever, headache, malaise, anorexia, nausea, poorly localized abdominal discomfort, a dry cough, and myalgia are all signs of typhoid fever [12].

Even though both malaria and typhoid fever are caused by different pathogens with different transmission modes, individuals are at risk of contracting both infections [13]. They equally share similar signs and symptoms [14]. The COVID-19 pandemic caused disruptions in healthcare systems, leading to a weakening of malaria surveillance efforts [15]. Malaria and typhoid fever have similar symptoms that lead clinicians to suspect both, often recommending dual tests. However, cross-reactions with the Widal and malaria rapid diagnostic tests (RDTs) can inflate prevalence estimates in epidemiological studies. Understanding the burden of these diseases and their risk factors is crucial for better prevention, care, and treatment. Despite the already existing data on malaria and typhoid fever in Cameroon, data on their prevalence in Yaoundé, Cameroon, are lacking. This study addresses this gap by investigating the prevalence of malaria, typhoid fever, and coinfection among febrile patients in Yaoundé, along with associated predictors for clinical–epidemiologic purposes.

## 2. Methods

### 2.1. Study Site

The study was conducted at the Etoug-Ebe Baptist Hospital, a major referral hospital in Yaoundé, located at 3° 50⁣′ 58.6⁣^″^ N and 11° 28⁣′ 59.6⁣^″^ E (latitude: 3.8496017, longitude: 11.4832077). Yaoundé is in the center region of Cameroon, lying within the rainforest belt of Central Africa and has a Guinea-type equatorial climate [16]. It is the political capital of Cameroon, with an estimated population of 2.5 million people over an area of 304 km^2^ and situated above sea level [17, 18]. The transmission of malaria in Yaoundé is perennial, and there are four distinct seasons: two rainy seasons (March–May/June and September–November) and two dry seasons (December–February and June/July–August) [17]. *Anopheles gambiae* and *Anopheles funestus* contribute to malaria transmission in urban Yaoundé, and their distribution is seasonal [18].

### 2.2. Study Population, Sample Size, and Design

This cross-sectional study was conducted between June and August 2023. Two hundred and eighty-eight febrile patients aged over 2 years who presented with a temperature of ≥ 37.5°C or a history of fever lasting more than 1 day were enrolled. Clinically suspected of malaria and/or typhoid fever, these patients were randomly selected from both inpatients and outpatients at the Etoug-Ebe Baptist Hospital. The sample size was calculated based on the prevalence of another study [14], using the formula described by Daniel and Cross (1999):

 $$\begin{array}{c} N=\frac{Z^{2}p\left(1-p\right)}{d^{2}} \end{array}$$

where

• *N* = required sample size,
• *Z* = statistic for a level of confidence (1.96 for 95% confidence),
• *p* = estimated prevalence in the study area,
• *d* = margin of error (5%).

Using the prevalence of malaria reported in Yaoundé (*p* = 28.65%) [14], the required sample size was 313 participants. Similarly, based on the prevalence of typhoid fever (*p* = 16.29%) [14], the required sample size was 212 participants.

On average, the calculated sample size was 262 febrile patients. To account for a potential 10% dropout rate, a total of 288 participants were finally recruited for this study.

### 2.3. Sample Collection and Biodata of Participants

Data were collected by the principal investigator and a trained technician. Prior to sample collection, informed consent was obtained from adult participants or from legal guardians in the case of minors, following ethical approval from the relevant ethics committees. Sociodemographic characteristics—including age, sex, residence, level of education, marital status, and occupation—were recorded, along with clinical signs and symptoms (type and severity), clinical history, and treatment approaches. Environmental data and behavioral factors potentially associated with malaria–typhoid infections were also collected using a pretested structured questionnaire. This questionnaire was administered through face-to-face interviews using a standardized case report form (CRF).

Following the interviews, approximately 3 mL of venous blood was collected aseptically using EDTA vacutainer tubes, and an additional 2 mL was collected in plain vacutainer tubes for serum separation. Whole blood samples were centrifuged at 3000 rpm for 5 min to separate plasma or serum prior to diagnostic testing. Also, stool samples were obtained from consenting participants using sterile, leak-proof stool collection containers. Samples were collected only from patients who were able to provide stool. To minimize contamination, participants were instructed on proper collection procedures, and all specimens were handled under aseptic conditions. For participants who could not provide a stool sample, typhoid fever diagnosis was performed only using a rapid typhoid diagnostic test with serum.

#### 2.3.1. Diagnostic of Malaria

##### 2.3.1.1. RDT for Screening of Malaria Parasite

Malaria diagnosis employed a commercially available RDT, the SD Bioline Malaria Ag Pf/Pan (Catalog Number 05FK40; sensitivity: 99.7%, specificity: 99.5%). This immunochromatographic assay detects *Pf* histidine-rich Protein 2 (HRP2) antigen and pan–malarial lactate dehydrogenase (pLDH) in finger-prick whole blood samples. Following the manufacturer's instructions, 5 *μ*L of EDTA-anticoagulated blood was dispensed into the sample well, followed by the addition of the designated diluent. After a 20-min incubation, results were interpreted based on the presence or absence of red lines in the control (C) and test (Pf or Pan) regions. A positive test displayed red lines in both the C and Pf or Pan regions. A negative result was indicated by a single red line in the C region only, while the absence of any red line in the C region signified an invalid test. All procedures were strictly adhered to as per the manufacturer's established protocols.

##### 2.3.1.2. Thick Blood Film for the Confirmation of Malaria Parasites

From each patient, a thick blood film was prepared using an EDTA blood sample. Thick blood smears were prepared and stained using the Giemsa staining technique as described elsewhere [19]. The Giemsa solution was filtered and diluted with distilled water before being applied to the blood film, which was then stained for 10 min and washed off. Slides were air-dried, examined under a microscope by a qualified microscopist, and deemed “negative” if no parasites were seen in at least 100 microscopic fields. Positive slides and 10% of negative slides were re-examined for quality control. Results were reported as the average number of parasites per 200/500 white blood cells, converted to parasites per microliter using the patient's white blood cell count or assuming a total of 8000 white blood cells. Parasitemia was calculated using a specific formula used in other studies [20]:

 $$\begin{array}{c} \text{Parasitemia}:\frac{\text{number of parasites}*8000}{\text{set range of WBCs }\left(200/500\right)}. \end{array}$$

For malaria diagnosis, we applied a combined diagnostic algorithm to ensure accuracy and precision during the determination of the prevalence. A case was considered malaria-positive if either of the following criteria were met: (1) a positive RDT confirmed by a positive thick blood smear or (2) a negative RDT followed by a positive thick blood smear. Only participants with both a negative RDT and a negative thick blood smear were considered malaria-negative. Results were reported based on this combined diagnostic outcome rather than per individual test.

However, it should be noted that not all participants underwent RDT testing due to occasional stockouts or clinical discretion. (3) In such cases, diagnosis relied solely on thick blood smear microscopy, which served as the primary diagnostic method and reference standard.

#### 2.3.2. Diagnosis of Typhoid Fever

##### 2.3.2.1. Widal Test

The Widal test qualitative and quantitative agglutination methods were performed with serum using the Felix Widal agglutination kit (BIOLABO SA, France; 70% specificity and 70% sensitivity) as described elsewhere [21]. This test includes coated somatic (O) and flagella (H) antigens for *S. typhi* and *S. paratyphi A*, *B*, and *C*. The O and H antigens suspension bottles represented antigens of *S. typhi*, while the AO, BO, CO, AH, BH, and CH antigens suspension bottles represent *S. paratyphi A*, *B*, and *C* species. All positive results with the slide method were confirmed and quantified by the tube agglutination method. The serum was tested at a dilution of 1/20, 1/40, 1/80, 1/160, and 1/320, and an antibody titer with a cutoff of ≥ 1/80 was considered positive for typhoid fever, as described elsewhere [8, 22, 23].

To complement the diagnosis of typhoid fever, two other rapid typhoid tests were used: one for antigens in stool and another for antibodies IgM/IgG in blood.

##### 2.3.2.2. Rapid Typhoid Test (Typhidot IgM/IgG)

The *AllTest* Rapid Typhoid IgM/IgG test (IT7-402, Hysen Hangzhou Biotech Co. Ltd; sensitivity: 93.9% [IgM]/86.7% [IgG] and specificity: 99% [IgM]/99.6% [IgG]) was used. It is a lateral flow chromatographic immunoassay that can detect and differentiate IgG and IgM antibodies to *S. typhi* or *S. paratyphi* in human blood through visual color development. During testing, 50 *μ*L of whole blood was added to the sample well, followed by one drop of sample diluent, and results were available in 15 min. The test was considered “positive” if a colored band appeared in the C line and another in the IgM or IgG line for IgM and IgG antibodies, respectively; meanwhile, “negative” was the absence of a colored band in the IgG and IgM lines.

##### 2.3.2.3. Rapid Typhoid Test for *Salmonella* O and H Antigens in Stool

The rapid typhoid test for *Salmonella typhoid* antigen (ISTY-602, Hysen Hangzhou Biotech Co. Ltd; 96.2% sensitivity and 98.3% specificity) was used. It is a qualitative lateral flow chromatographic immunoassay for the detection of *Salmonella* antigen in fecal samples. During testing, antibodies of *Salmonella* present in the sample well react with *Salmonella* antigens present in the stool. Around 160 mg/mL of the fecal specimen was introduced into the buffer vial and shaken vigorously to mix the specimen and the extraction buffer. The cap of the vial was broken off, and two drops were dispensed into the specimen well. The result was read 10 min after dispensing the sample. A colored test and C line indicated a positive result for *Salmonella* antigen, and a colored C line indicated a negative result.

For typhoid fever diagnosis, a stepwise diagnostic algorithm was used to enhance diagnostic accuracy. A participant was classified as typhoid fever-positive if the following criteria were met: (1) a positive Widal tube agglutination test (antibody titer ≥ 1/80), clinical signs and symptoms suggestive of typhoid fever, and at least one positive result from a rapid typhoid test, either for antibodies (IgM/IgG) or for stool antigens. (2) A case was considered typhoid fever-negative if both the Widal test and the rapid typhoid tests (antibody and antigen detection) were negative. (3) In case the RTT result was not available, priority was given to the Widal tube agglutination test combined with clinical assessment.

### 2.4. Data Analysis

Data generated were entered into Microsoft Office Excel 2021, and the data were exported and analyzed using SPSS statistical software, Version 26.0. In this study, a *p* value < 0.05 was considered significant at a 95% CI. Graphs and figures were created using GraphPad (GraphPad Prism, Version 9.00, La Jolla, California, United States). To determine the prevalence of malaria and typhoid fever and the coinfection in the study population, an exploratory data analysis (EDA) was performed. Categorical variables were presented as frequencies and percentages. The chi-square test or Fisher's exact *t*-test (in case of 20% of categories with expected frequencies less than 5) was used to compare group differences between categorical variables. A multivariate logistic regression was used to determine associated risk factors for malaria and typhoid fever.

### 2.5. Ethical Consideration

The ethical clearance was obtained from the Centre Regional Ethics Committee for Human Health Research CRERSH-Ce (Ref #: 0226-CRERSHC/2022) with administrative authorization from the Regional Delegation of Public Health (Ref #: 1392-7/AAR/MINSANTE/SG/DRSPC) and the Institutional Review Board of the Cameroon Baptist Convention (Ref #: IRB2023-26). The research was carried out in compliance with current national (law n° 2022-008 of the 27th April 2022) and international regulations as the declaration of Helsinki (2013). The relevant ethics boards and regulatory authorities approved the protocol before the study's commencement. The information sheet and consent form were available in English and French. The form was given to the patient or their representative and carefully explained verbally. Adults who agreed to participate in the study, representatives of children who consented to their child's participation, and representatives of adults with impaired consciousness who consented to the patient's participation were asked to sign the form.

## 3. Results

### 3.1. Sociodemographic Characteristics

A total of 288 febrile patients aged more than 2 years old attending the Etoug-Ebe Baptist Hospital for consultation and suspected to have malaria and/or typhoid fever were included in this study. The majority of participants were females (*n* = 161, 55.09%). The study population was predominantly composed of individuals aged over 15 years (*n* = 233, 80.9%), with a mean age of 28.25 years. Two other age groups were represented to a lesser extent: children aged 2–5 years (*n* = 20, 6.9%), considered at higher risk for malaria, and children aged 6–15 years (*n* = 35, 12.2%), typically at risk for typhoid fever. Together, these two younger age groups accounted for less than 20% of the total study population. The fever duration varied from 1 to 2 months, with a mean temperature of 37.39°C. The majority of participants identified as Christian (96.9%), while a minority identified as Muslim (3.1%). Most participants were private workers (44.8%) and living in urban areas (91.3%), and the majority had attended university (66.7%), with a small percentage being illiterate (2.8%) (Table 1). Only 117 participants (40.63%) had a body temperature of ≥ 37.5°C.

### 3.2. Prevalence of Malaria, Typhoid Fever, and the Coinfection

The study identified a high infection rate (over 62%) among febrile patients in Yaoundé, Cameroon. Malaria emerged as the most prevalent disease (44.8%); the prevalence of malaria only was 25% (*n* = 72), and males were more infected than females (52.77%). Parasitemia levels ranged from 8 to 120,000. A total of 86 subjects (29.9%) had counts below 500, 24 (8.3%) had counts between 501 and 5000, 18 (6.3%) fell within the range of 5001–500,000, and 3 subjects (1%) had counts up to 500,000 parasites.

Using our diagnostic workflow, we confirmed that the overall prevalence of typhoid fever was 37.15% among participants with typhoid fever or coinfection, and 17.36% among those with typhoid fever only. Of the participants with typhoid fever, 44% (*n* = 22) were male and 56% (*n* = 28) were female. By the Widal test, *S. typhi* and *S. paratyphi B* were the most common causes of typhoid fever, accounting for 35 cases each. *S. typhi* was more likely to coexist with other species, with 23 cases of mixed infections and 12 cases of single infections. *S. paratyphi A* was present in 26 cases (24.29%), *S. paratyphi C* in 8 cases (7.37%), and there were three cases of mixed infections with other species. Interestingly, *S. typhi* and *paratyphi B* were often found to coexist with malaria infections. A coinfection prevalence of 19.79% (*n* = 57) was observed. Females were more infected than males (Table 2).

Before seeking medical consultation at the hospital, patients reported self-medicating. Among those who had taken medication before admission, the most common types of medication were antimalarials (10.1%), antibiotics (2.8%), antipyretics (18.1%), and vitamins (3.2%). Additionally, some patients used traditional medicine (2.8%) (Table 3). However, the majority of participants (63.2%) had not taken any medication before consulting the healthcare facility. Interestingly, our analysis found an association between not taking any medication before admission and a diagnosis of typhoid fever (*p* = 0.039) (Table 3).

### 3.3. Risk Factors and Determinants Associated With Malaria and Typhoid Fever

Sociodemographic characteristics, such as age, sex, occupation, and education level, did not show a significant association with malaria and typhoid fever (*p* = 0.049). In terms of clinical features, jaundice was found to be significantly associated with typhoid fever (*χ*^2^ = 4.77, *p* = 0.029). Additionally, profuse sweating was slightly significant with coinfection (*χ*^2^ = 3.774, *p* = 0.049) (Table 4). Furthermore, there was a significant association between fever (with a temperature of ≥ 37.5°C) and coinfection (*p* = 0.008). Using multivariate logistic regression analysis, the presence of water and/or clumps of grass around the house was a significant risk factor for malaria (AOR = 1.972, *p* = 0.018, 95% CI: [1.12–3.43]) (Table 5). For typhoid fever, tap water as a source of drinking water was a significant risk factor (AOR: 4.29, *p* = 0.04, 95% CI: [1.01–18.18] (Table 6).

## 4. Discussion

Reports have identified malaria and typhoid fever among the most significant infectious diseases affecting the African population [22]. Early diagnosis and proper treatment of infectious diseases should be employed to reduce disease severity, burden, resistance, and mortality. Many epidemiological studies have already been conducted in Cameroon [12, 14, 17, 23–27] and in other countries in Africa such as Nigeria [28–31], Ethiopia [8, 32], and Ghana [22, 33, 34]. Given the evolving public health landscape, particularly in the post-COVID-19 context, this study is aimed at updating existing data on the prevalence and dynamics of malaria and typhoid fever in Yaoundé, Cameroon. By identifying potential risk factors associated with these diseases, patient care and management strategies could be enhanced. Understanding the current situation can inform interventions tailored to the specific challenges faced in this region, ultimately contributing to better health outcomes for affected populations.

### 4.1. Prevalence of Malaria and Risk Factors

**Figure 1:**
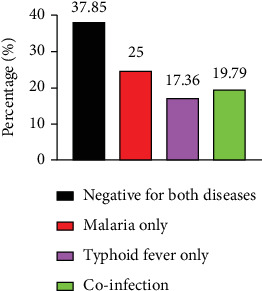
Distribution of malaria and typhoid fever among febrile patients.

Out of the 288 participants recruited for this study, Figure 1 reports malaria; it is the most frequently detected infection. This finding aligns with previous reports by Nsutebu et al. [26] among feverish adults and Achonduh-Atijegbe et al. [17] among feverish children in Yaoundé-Cameroon, but differs from what was published recently in Ngaoundéré, with typhoid fever being the most reported infection [25]. This discrepancy may be attributable to geographical differences and seasonal factors, as malaria transmission can vary throughout the year, and the timing of studies can significantly influence results.

This prevalence of malaria (Figure 1) is comparable to rates reported in Nigeria and Ghana, which range between 36% and 40% [13, 34]. Nonetheless, it is lower than the 50% prevalence reported in northern Cameroon [25] but higher than findings from other regions in Cameroon, such as 10.3% in Fondonera [27], 19.4% in Douala [12], and 24.2% in Northern Tanzania [35]. The variation in prevalence rates among different studies could be due to climatic differences, seasonal variations, and potentially different testing methodologies. The disruption of malaria surveillance and healthcare services during the COVID-19 pandemic in Yaoundé, along with human migration patterns and the emergence of resistance to antimalarial drugs, are plausible factors contributing to the higher prevalence observed in this study. These findings underscore a potential breakdown in malaria control strategies within this locality. Also, the malaria prevalence obtained in this study is well above the national malaria prevalence (44.79% vs. 30%) [36], indicating an urgent need to strengthen malaria control protocols. Enhanced surveillance, targeted interventions, and resource allocation are essential to address the rising burden of malaria in Yaoundé and improve overall public health outcomes.

Table 2 presents the association between sociodemographic characteristics and malaria infection. Our findings are consistent with studies conducted in Ethiopia and Ghana [8, 34]; however, other research has reported an association between gender and malaria infection [25]. In Table 5, multivariate logistic regression reveals that ponds of water and/or clumps of grass around the house was a significant risk factor (AOR = 1.97, *p* = 0.018, 95% CI: [1.12–3.43]). This highlights the ongoing risk of malaria transmission in Yaoundé, indicating a pressing need for effective strategies to mitigate the burden of this disease. Public education campaigns on the importance of sleeping under treated mosquito nets and maintaining clean surroundings around homes are essential. Additionally, there is a need for continuous monitoring and effective distribution of insecticide-treated nets throughout Yaoundé, Cameroon, to enhance preventive measures against malaria.

### 4.2. Prevalence of Typhoid Fever and Risk Factors

Figure 1 presents the prevalence of typhoid fever in this study, and the overall prevalence is 37.15% (*n* = 107). This prevalence is higher compared to what was reported in some parts of Cameroon and beyond, such as in Yaoundé (2.5%) [26], Douala (32.5%) [12], Buea (7.9%) [25], and west Ghana (14.36%) [22]. However, it is quite lower compared to many previous reports in northern Cameroon (64.3%) [25]. The higher prevalence of typhoid fever observed in this study may be attributed to a lack of attention to water and food safety among residents of Yaoundé. Contributing factors include inadequate sanitation infrastructure and poorly maintained toilet facilities throughout the city. The student population, particularly those aged 12–25, exhibited the highest rates of infection, although this finding was not statistically significant. This demographics' active social engagement increases their exposure risk; many consume commercially bottled water, practice inadequate hand hygiene, and purchase food from street vendors. These behaviors may collectively contribute to the elevated prevalence of typhoid fever in this locality, as highlighted elsewhere [12–25].

**Figure 2:**
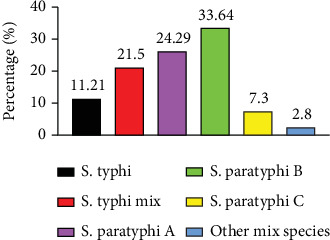
Repartition of *Salmonella* species in the study population.

Using the Widal test, *S. typhi* and *S. paratyphi B* were the most common species (35/107 cases) (Figure 2), and *S. typhi* was most likely to coexist with other *Salmonella* bacteria. These results align with the findings of Njolle et al. and Netongo et al., who reported *S. typhi* as the predominant species [12, 14], followed by *S. paratyphi B* [12]. There is an important contribution of *S. typhi* and *paratyphi B* to the burden of febrile illness in the area.

In Table 4, an association with jaundice was found to be significant. Typhoid hepatitis results from the invasion of the liver by *Salmonella*, which leads to damage to hepatocytes and induces the synthesis and release of cytokines. Consequently, jaundice can serve as a clinical marker for the suspicion of typhoid fever in patients presenting with fever, as noted by other authors [37, 38]. Tap water consumption was identified as a potential risk factor for typhoid fever in our study population (AOR = 4.29, *p* = 0.04, 95% CI: [1.01–18.18]) (Table 6). This underscores the urgent need to strengthen typhoid fever control measures. Public education on good hygienic practices and the availability of safe water to the population is essential in Yaoundé, Cameroon, to mitigate the incidence of this disease.

### 4.3. Malaria and Typhoid Fever Coinfection

Figure 1 represents the prevalence of malaria and typhoid fever coinfection. Netongo et al. reported a prevalence of 13.48% malaria and typhoid fever coinfection [14] in the same population. The higher rate of coinfection recorded here could be due to the diagnostic tests used, differences in sample size, seasonal changes, and other related human factors. It is crucial to employ appropriate diagnostic tests when a patient presents with signs and symptoms suggestive of either malaria or typhoid fever to minimize diagnostic confusion.

In Table 4, an association between profuse sweating, fever (with temperatures exceeding 37.5°C), and the coinfection status was established, corroborating the findings of Alelign et al. and Chilongola et al. [32, 35]. These results suggest that fever and chills may serve as valuable clinical indicators of coinfection in febrile patients. Findings indicated that *Plasmodium* is likely to coexist with *S. typhi* (*n* = 18/57) and *S. paratyphi* B (*n* = 18/57). It is well established that hemolysis, resulting from the degradation of red blood cells, leads to the release of iron, which is a vital element required for the growth of *Salmonella* [3].

### 4.4. Treatment Approaches Before Admission

As shown in Table 3, the self-medication rate in this study population was relatively low at 36.8%, particularly when compared to a previous study conducted among children in Yaoundé, Cameroon [17]. This self-medication could potentially contribute to the development of antibiotic and antimalarial resistance, complicating effective treatment. Despite the lower rate of self-medication observed in this study, there is still a pressing need for ongoing education within the community regarding the risks associated with self-medication. This information should be incorporated into malaria and typhoid fever control programs. The use of medicinal plants before seeking medical consultation for fever may be attributed to poverty and underdevelopment, particularly in low- and middle-income countries [25].

## 5. Conclusions

This study highlights malaria as the leading cause of febrile illness among patients in Yaoundé, with a notable prevalence of malaria–typhoid coinfection, which may be influenced by overdiagnosis or local environmental conditions. Typhoid fever showed a significant association with jaundice, while coinfections correlated with symptoms such as sweating and fever. Identified environmental risk factors, including stagnant water, overgrown vegetation near households, and unsafe drinking water sources, further contribute to the burden of these infections.

These findings call for targeted interventions to strengthen malaria and typhoid fever control strategies in Yaoundé. Public health authorities and stakeholders should prioritize improved community education on good hygienic practices and the importance of seeking appropriate healthcare. Additionally, integrating routine environmental management with comprehensive screening for febrile illnesses beyond malaria could enhance diagnostic accuracy and treatment outcomes. Implementing these recommendations will support evidence-based policy decisions and help reduce the burden of febrile diseases in this region.

## Figures and Tables

**Table 1:** Sociodemographic characteristics of study participants at the Etoug-Ebe Baptist Hospital, Yaoundé, Cameroon (*N* = 288).

**Demographic characteristics**	**Frequencies (** **N** **)**	**Percentage (%)**
Age group		
[02–05]	20	6.9
[06–15]	35	12.2
> 15	233	80.9
Religion		
Muslim	9	3.1
Christian	279	96.9
Gender		
Male	127	44.1
Female	161	55.9
Profession		
Unemployed	31	10.8
Student	110	38.2
Public worker	18	6.3
Private worker	129	44.8
Level of education of the participant		
Illiterate	8	2.8
Nursery school	11	3.8
Primary school	20	6.9
Secondary school	57	19.8
University	192	66.7
Marital status		
Widow	5	1.7
Single	183	63.5
Fiancé	2	0.7
Married	98	34
Residence		
Subrural	25	8.7
Urban	263	91.3

**Table 2:** Prevalence of malaria, typhoid fever and the co-infection in relation to Socio-demographics Characteristics among fever patients attending the Etoug-Ebe Baptist Hospital, Yaoundé-Cameroon.

**Variables**	**Malaria**				**Typhoid fever**				**Coinfection**			
	**Positive ** **N** ** (%)**	**Negative ** **N** ** (%)**	**χ** ^2^	**p** ** value**	**Positive ** **N** ** (%)**	**Negative ** **N** ** (%)**	**χ** ^2^	**p** ** value**	**Positive ** **N** ** (%)**	**Negative ** **N** ** (%)**	**χ** ^2^	**p** ** value**
Gender												
Male	38 (29.9)	89 (70.07)	0.38	0.54	22 (17.3)	105 (82.7)	0.00	0.98	22 (17.3)	105 (82.7)	0.87	0.35
Female	34 (21.1)	127 (78.8)			28 (17.4)	133 (82.6)			35 (21.7)	126 (78.3)		
Age group												
[2–5]	4 (20)	16 (80)	0.35	0.84	2 (10)	18 (90)	1.99	0.37	1 (5)	19 (95)	4.46	0.1
[6–15]	8 (11.3)	27 (77.1)			4 (11.4)	31 (88.6)			10 (28.6)	25 (71.4)		
>15	60 (25.75)	173 (74.25)			50 (50)	189 (81.1)			46 (19.7)	187 (80.3)		
Residence												
Rural	7 (28)	18 (72)	0.16	0.68	3 (12)	22 (20.7)	0.55	0.46	8 (32)	17 (68)	2.3	0.11
Urban	65 (24.71)	198 (75.29)			47 (17.9)	216 (82.1)			49 (18.6)	214 (81.4)		
Profession												
Unemployed	5 (16.1)	26 (83.9)	1.5	0.68	6 (19.4)	25 (80.6)	1.25	0.7	6 (19.4)	25 (80.6)	0.37	0.95
Student	29 (26.4)	81 (73.6)			22 (20)	88 (80)			20 (18.2)	90 (81.8)		
Public worker	4 (22.2)	14 (77.8)			3 (16.7)	15 (83.3)			4 (22.2)	14 (77.8)		
Private worker	33 (25.6)	96 (74.4)			19 (14.7)	110 (85.3)			27 (20.9)	102 (79.1)		
Educational status^a^												
Illiterate	2 (25)	6 (75)	7.16	0.128	1 (12.5)	7 (87.5)	4.13	0.356	0 (0.0)	8 (100)	2.91	0.569
Nursery school	2 (18.2)	9 (81.8)			1 (9.1)	10 ((90.9)			1 (9.1)	10 (90.9)		
Primary school	4 (20)	16 (80)			2 (10)	18 (90)			4 (20)	16 (80)		
Secondary school	22 (38.6)	35 (61.4)			6 (10.5)	51 (89.5)			14 (24.6)	43 (75.4)		
University	42 (21.9)	150 (78.1)			40 (20.8)	152 (79.2)			38 (19.8)	154 (80.2)		
Matrimonial status^a^												
Window	1 (20)	4 (80)	1.65	0.658	0 (0.0)	5 (100)	0.95	0.738	4 (80)	1 (20)	**10.2**	**0.012**
Single	48 (26.2)	135 (73.8)			31 (16.9)	152 (83.1)			31 (16.9)	152 (83.1)		
Fiancé	1 (50)	1 (50)			0 (0.0)	2 (100)			0 (0.0)	2 (100)		
Married	22 (22.4)	76 (77.6)			19 (19.4)	79 (81)			22 (22.4)	76 (77.6)		

*Note:* Bold numbers represent significant association test.

^a^Fisher's exact *t*-test performed, 20% of the expected count was less than 5.

**Table 3:** Distribution of malaria, typhoid fever, and the coinfection in patients according to preadmission treatment.

**Treatment approach**	**Malaria**				**Typhoid fever**				**Coinfection**			
	**Positive ** **N** ** (%)**	**Negative ** **N** ** (%)**	***χ*** **^2^**	**p** ** value**	**Positive ** **N** ** (%)**	**Negative ** **N** ** (%)**	***χ*** **^2^**	**p** ** value**	**Positive ** **N** ** (%)**	**Negative ** **N** **(%)**	***χ*** **^2^**	**p** ** value**
Antimalarial												
Yes	7 (24.1)	22 (75.9)	0.005	0.9	4 (13.8)	25 (86.2)	0.28	0.59	6 (20.7)	23 (79.3)	0.016	0.9
No	65 (25)	195 (75)			46 (17.8)	213 (82.2)			51 (19.7)	208 (80.3)		
Antibiotics												
Yes	3 (37.5)	5 (62.5)	0.73	0.39	0 (0.0)	8 (100)	1.73	0.19	2 (25)	6 (75)	0.14	0.71
No	69 (24.6)	212 (75.4)			50 (17.9)	230 (82.1)			55 (19.6)	225 (80.4)		
Traditional medicine												
Yes	3 (37.5)	5 (62.5)	0.73	0.39	1 (12.5)	7 (87.5)	0.14	0.71	2 (25)	6 (75)	0.14	0.1
No	69 (24.6)	212 (75.4)			49 (17.5)	231 (82.5)			55 (19.6)	225 (80.4)		
Antipyretics												
Yes	16 (30.8)	36 (69.2)	1.3	0.26	7 (13.5)	45 (86.5)	0.67	0.41	8 (15.4)	44 (84.6)	0.78	0.38
No	56 (23.6)	181 (76.4)			43 (18.2)	193 (81.8)			49 (20.8)	187 (79.2)		
Others (vitamins)												
Yes	1 (11.1)	8 (88.9)	0.1	0.34	1 (11.1)	8 (88.9)	0.25	0.62	3 (33.3)	6 (66.7)	1.07	0.3
No	71 (25.4)	209 (74.6)			49 (17.6)	230 (82.4)			54 (19.4)	225 (80.6)		
No medication												
Yes	40 (22)	142 (78)	1.9	0.17	38 (20.9)	144 (79.1)	4.26	**0.039**	35 (19.2)	147 (80.2)	0.1	0.75
No	32 (29.9)	75 (70.1)			12 (11.3)	94 (88.7)			22 (20.8)	84 (79.2)		

*Note:* Bold numbers represent significant association test.

**Table 4:** Prevalence of malaria, typhoid fever, and the coinfection according to signs and symptoms among febrile patients attending the Etoug-Ebe Baptist Hospital, Yaoundé, Cameroon.

**Variables**	**Malaria**				**Typhoid fever**				**Coinfection**			
	**Positive ** **N** ** (%)**	**Negative ** **N** ** (%)**	***χ*** **^2^**	**p** ** value**	**Positive ** **N** ** (%)**	**Negative ** **N** ** (%)**	***χ*** **^2^**	**p** ** *value***	**Positive ** **N** ** (%)**	**Negative ** **N** ** (%)**	***χ*** **^2^**	**p** ** value**
Fever (with temperature ≥ 37.5)												
Yes	29 (24.8)	88 (75.2)	0.005	0.95	22 (18.8)	95 (81.2)	0.286	0.59	32 (27.4)	85 (72.6)	7.092	**0.008**
No	43 (25.1)	128 (74.9)			28 (16.4)	143 (83.6)			25 (14.6)	146 (85.4)		
Headache												
Yes	56 (25.8)	161 (74.2)	0.305	0.581	39 (78)	178 (82)	0.229	0.632	46 (21.2)	171 (78.8)	1.097	0.295
No	16 (22.5)	55 (77.5)			11 (22)	60 (84.5)			11 (15.5)	60 (84.5)		
Abdominal pain												
Yes	24 (24.2)	75 (75.8)	0.046	0.830	21 (21.2)	78 (78.8)	1.559	0.212	24 (42.1)	75 (75.8)	1.882	0.170
No	48 (25.2)	141 (74.6)			29 (15.3)	160 (84.7)			33 (17.5)	156 (82.5)		
Shaking chill^a^												
Yes	1 (16.7)	5 (83.3)	0.227	0.634	1 (16.7)	5 (83.3)	0.002	1.000	1 (1.8)	5 (83.3)	0.038	1.000
No	71 (25.2)	211 (74.8)			49 (17.4)	238 (82.6)			56 (19.9)	226 (80.1)		
Anorexia												
Yes	7 (28)	18 (72)	0.131	0.717	3 (12)	22 (88)	0.548	0.459	4 (216)	21 (84)	0.248	0.619
No	65 (24.7)	198 (75.3)			47 (17.9)	216 (82.1)			53 (20.2)	210 (79.8)		
General body pain												
Yes	16 (21.1)	60 (78.9)	0.858	0.354	18 (23.7)	58 (76.3)	2.877	0.09	19 (25)	57 (75)	1.764	0.184
No	56 (26.4)	156 (73.6)			32 (36.8)	180 (84.9)			38 (17.9)	174 (82.1)		
Jaundice^a^												
Yes	0 (0.0)	1 (1000	0.334	1.000	1 (100)	0 (0.0)	4.777	**0.029**	0 (0.0)	1 (100)	0.248	1.000
No	72 (25.1)	215 (74.9)			49 (17.1)	238 (82.9)			57 (19.9)	230 (80.1)		
Profuse sweating^a^												
Yes	5 (41.7)	7 (58.3)	1.855	0.182	1 (8.3)	11 (91.7)	0.711	0.698	5 (41.7)	7 (58.3)	3.774	**0.049**
No	67 (24.3)	209 (75.7)			49 (17.8)	227 (82.2)			52 (18.8)	224 (81.2)		
Vomiting/nausea												
Yes	12 (24.5)	37 (75.5)	0.008	0.928	8 (16.3)	41 (83.7)	0.044	0.834	10 (17.5)	39 (79.6)	0.014	0.905
No	60 (25.1)	179 (74.9)			42 (17.6)	197 (82.4)			47 (19.7)	192 (80.3)		
Diarrhea^a^												
Yes	1 (25)	3 (75)	0.000	1.000	0 (0.0)	4 (100)	0.852	1.000	2 (50)	2 (50)	2.322	0.177
No	71 (98.6)	213 (75)			50 (49.3)	234 (82.4)			55 (19.4)	229 (80.6)		

*Note:* Bold numbers represent significant association test.

^a^Fisher's exact *t*-test performed, 20% of the expected count was less than five.

**Table 5:** Determinants associated with malaria infection among febrile patients at the Etoug-Ebe Baptist Hospital, Yaoundé, Cameroon.

**Variables**	**Positive ** **N** ** (%)**	**Negative ** **N** ** (%)**	**p** ** value**	**AOR**	**95% CI**
Gender					
Male	38 (29.9)	89 (70.07)	0.404	0.785	[0.445–1.385]
Female	34 (21.1)	127 (78.8)	/	1	
Age group					
[2–5]	4 (20)	16 (80)	0.924	0.94	[0.28–3.145]
[6–15]	8 (11.3)	27 (77.1)	0.96	1.6	[0.4–2.33]
> 15	60 (25.75)	173 (74.25)	/	/	
Residence					
Subrural	7 (28)	18 (72)	0.651	0.804	[0.313–2.07]
Urban	65 (24.71)	198 (75.29)	/	1	
Sleep under a mosquito net					
Yes	33 (24.8)	100 (75.2)	/	1	[0.516–1.65]
No	39 (25.2)	116 (74.8)	0.787	0.923	
Ponds of water and/or clumps of grass around the house					
Yes	34 (33)	69 (67)	**0.018**	**1.972**	[1.12–3.431]
No	38 (22.6)	147 (77.4)	/	1	

*Note:* Bold numbers represent significant association test.

**Table 6:** Determinants associated with typhoid fever among febrile patients attending the Etoug-Ebe Baptist Hospital, Yaoundé, Cameroon.

**Variables**	**Positive ** **N** ** (%)**	**Negative ** **N** ** (%)**	**p** ** value**	**AOR**	**95% CI**
Gender					
Male	22 (17.3)	105 (82.7)	/	1	
Female	28 (17.4)	133 (82.6)	0.65	1.6	[0.21–12.28]
Age group					
[2–5]	2 (10)	18 (90)	/	1	
[6–15]	4 (11.4)	31 (88.6)	0.65	1.6	[0.21–12.3]
> 15	50 (50)	189 (81.1)	0.35	2.26	
Main source of drinking water					
Mineral water	7 (11.7)	53 (88.3)	/	1	
Borehole	35 (17.8)	162 (82.2)	0.18	1.925	[0.73–5.07]
Spring	3 (21.4)	11 (78.6)	0.24	2.71	[0.52–14.15]
Tap water	5 (29.4)	12 (70.6)	**0.04**	**4.29**	[1.01–18.18]
Wash hands habits					
Yes	40 (20.3)	157 (79.7)	/	1	
Not always	10 (11)	81 (89)	**0.032**	**0.39**	[0.17–0.92]
Ice cream consumption					
Yes	10 (20)	40 (80)	/	1	
No	40 (16.8)	198 (83.2)	0.36	1.52	[0.62–3.73]
Drinks commercial water sold across the road					
Yes	20 (18.7)	87 (81.3)	0.46	1.44	[0.55–3.79]
No	30 (16.6)	151 (83.4)	/	1	
Toilet in home					
Yes	47 (18.8)	203 (81.2)	0.06	0.29	[0.08–1.053]
No	4 (7.9)	35 (92.1)	/	1	
Place of eating					
In house	26 (16.3)	134 (83.8)	/	1	
In house and outside (street food stall and restaurant)	24 (18.8)	104 (81.3)	0.49	1.27	[0.63–2.53]
Eat raw vegetables/fruits					
Yes	39 (16.3)	201 (83.8)	0.052	0.42	[0.17–1.00]
No	11 (22.9)	37 (77.1)	/	1	
Residence					
Subrural	3 (12)	22 (20.7)	0.28	2.08	[0.55–7.93]
Urban	47 (17.9)	216 (82.1)	/	1	

*Note:* Bold numbers represent significant association test.

## Data Availability

The data collected in this study are available upon request from the corresponding author.
